# Bioimpedance Alerts from Cardiovascular Implantable Electronic Devices: Observational Study of Diagnostic Relevance and Clinical Outcomes

**DOI:** 10.2196/jmir.8066

**Published:** 2017-11-23

**Authors:** Christophe JP Smeets, Julie Vranken, Jo Van der Auwera, Frederik H Verbrugge, Wilfried Mullens, Matthias Dupont, Lars Grieten, Hélène De Cannière, Dorien Lanssens, Thijs Vandenberk, Valerie Storms, Inge M Thijs, Pieter M Vandervoort

**Affiliations:** ^1^ Mobile Health Unit Faculty of Medicine and Life Sciences Hasselt University Hasselt Belgium; ^2^ Department of Cardiology Ziekenhuis Oost-Limburg Genk Belgium; ^3^ Future Health Department Ziekenhuis Oost-Limburg Genk Belgium

**Keywords:** defibrillators, implantable, cardiac resynchronization therapy, telemedicine, electric impedance, algorithms, call centers

## Abstract

**Background:**

The use of implantable cardioverter-defibrillators (ICDs) and cardiac resynchronization therapy (CRT) devices is expanding in the treatment of heart failure. Most of the current devices are equipped with remote monitoring functions, including bioimpedance for fluid status monitoring. The question remains whether bioimpedance measurements positively impact clinical outcome.

**Objective:**

The aim of this study was to provide a comprehensive overview of the clinical interventions taken based on remote bioimpedance monitoring alerts and their impact on clinical outcome.

**Methods:**

This is a single-center observational study of consecutive ICD and CRT patients (n=282) participating in protocol-driven remote follow-up. Bioimpedance alerts were analyzed with subsequently triggered interventions.

**Results:**

A total of 55.0% (155/282) of patients had an ICD or CRT device equipped with a remote bioimpedance algorithm. During 34 (SD 12) months of follow-up, 1751 remote monitoring alarm notifications were received (2.2 per patient-year of follow-up), comprising 2096 unique alerts (2.6 per patient-year of follow-up). Since 591 (28.2%) of all incoming alerts were bioimpedance-related, patients with an ICD or CRT including a bioimpedance algorithm had significantly more alerts (3.4 versus 1.8 alerts per patient-year of follow-up, *P*<.001). Bioimpedance-only alerts resulted in a phone contact in 91.0% (498/547) of cases, which triggered an actual intervention in 15.9% (87/547) of cases, since in 75.1% (411/547) of cases reenforcing heart failure education sufficed. Overall survival was lower in patients with a cardiovascular implantable electronic device with a bioimpedance algorithm; however, this difference was driven by differences in baseline characteristics (adjusted hazard ratio of 2.118, 95% CI 0.845-5.791). No significant differences between both groups were observed in terms of the number of follow-up visits in the outpatient heart failure clinic, the number of hospital admissions with a primary diagnosis of heart failure, or mean length of hospital stay.

**Conclusions:**

Bioimpedance-only alerts constituted a substantial amount of incoming alerts when turned on during remote follow-up and triggered an additional intervention in only 16% of cases since in 75% of cases, providing general heart failure education sufficed. The high frequency of heart failure education that was provided could have contributed to fewer heart failure–related hospitalizations despite significant differences in baseline characteristics.

## Introduction

Cardiac resynchronization therapy (CRT) devices and implantable cardioverter-defibrillators (ICDs) are guideline-recommended treatments for heart failure with reduced ejection fraction, left bundle branch block, very wide QRS complex (>150 ms), or sudden cardiac death [1,2]. The use of these cardiovascular implantable electronic devices (CIEDs) is rapidly increasing with 51,274 and 85,289 patients, respectively, receiving a CRT or ICD device in Europe in 2013 [3]. Remote follow-up of this group is slowly finding its way into routine clinical practice since it may hold major advantages for patients, health care workers, and society [4]. Over the last decade, several CIED manufacturers have marketed thoracic impedance measurement algorithms integrated into their devices. Changes in bioimpedance measurements reflect changes in intrathoracic fluid status and are evaluated based on a vendor-specific computer algorithm. Early investigations reported an inverse correlation with pulmonary capillary wedge pressure and fluid balance [5] and a higher sensitivity and lower unexplained detection rate compared to acute weight changes [6]. In addition, a decrease in bioimpedance happened even before clinical manifestation of heart failure worsening and before hospital admission for fluid overload [5]. These algorithms are therefore very promising for the early detection of impending decompensated heart failure and enable the possibility to adjust treatment strategies in order to prevent heart failure hospitalization [7,8]. Despite early investigations showing promising results, larger randomized trials have revealed disappointing outcomes [9-12]. A shortcoming in current studies is the lack of standardization and information as to what clinical actions are coupled to remote bioimpedance alerts, making it difficult to draw conclusions on its clinical impact.

In 2010, a dedicated remote follow-up program of heart failure patients with a CIED was started at Ziekenhuis Oost-Limburg (Genk, Belgium). Dedicated nurses, trained in electrophysiology, device follow-up, and heart failure pathophysiology, review all incoming alerts in a systematic and standardized manner with automatic interventions triggered by protocol-based, guideline-recommended care [13,14]. In this observational registry study, we closely analyzed all bioimpedance alerts and subsequent triggered interventions from OptiVol and OptiVol 2.0 (Medtronic PLC) and CorVue (St. Jude Medical LLC) CIEDs using the default alert settings, and we studied their impact on clinical outcome. As such, the current research builds on previous studies since these lack this level of detail.

## Methods

### Study Design

This is an observational registry study of ICD and CRT patients from a single tertiary care center (Ziekenhuis Oost-Limburg, Genk, Belgium) implanted with the devices between February 2010 and May 2013. Since February 2010, all patients receiving either an ICD or CRT device with remote monitoring capabilities were asked to participate voluntarily in a remote follow-up program. The type of CIED that was implanted was solely based on the device’s therapeutic capabilities (right ventricular pacing, biventricular pacing, and antitachycardia treatment) and was left to the discretion of the treating physician. For this analysis, only patients enrolled in remote follow-up within 6 months after device implantation are included. Patient baseline information is collected at the time of device implantation. All participants provided written informed consent and were followed until February 1, 2015. The study complies with the Declaration of Helsinki, and the study protocol was approved by the local committee on human research.

### Remote Follow-Up and Alerts

A vendor-specific transmission device, usually installed in the patient’s bedroom, collected disease- and device-related data from the CIED that was transmitted to an online database accessible to the multidisciplinary heart failure team. All alerts were interpreted on weekdays by dedicated nurses trained in electrophysiology, device follow-up, and heart failure pathophysiology; notifications received during weekends were read on Monday. Daily alert transmissions were generated when predefined alarm thresholds were crossed. Besides alert transmissions, each device was programmed to send a scheduled transmission report monthly. Alerts were categorized according to their nature into technical (missed scheduled transmission and technical device problems) or clinical (rhythm, bioimpedance, and miscellaneous [changes in daily activity, heart failure management, etc]) alerts. Our study focuses on all bioimpedance-related alerts.

### Bioimpedance Measurements

Since bioimpedance is measured from the electrode lead to the device can, any thoracic fluid change including vascular, interstitial, or alveolar fluid results in a change in its value. Therefore, bioimpedance measurements are not specific to one disease.

In 2004, Medtronic was the first company to introduce a bioimpedance algorithm, known as the OptiVol algorithm, in its CIEDs. For the OptiVol algorithm, bioimpedance is measured in a semicontinuous way every 20 minutes from 12 AM to 5 PM. The algorithm starts 34 days postimplant and generates 2 separate graphs: one displays the raw bioimpedance data and the other indicates the accumulated change between the daily bioimpedance measurements and a dynamic reference impedance. The latter one, called the OptiVol fluid index, triggers an alarm when a predefined threshold is met, by default set at 60Ω [5]. The OptiVol fluid index graph may indicate an event, while the raw bioimpedance graph may indicate the severity of the event [15,16]. In 2010, Medtronic launched OptiVol 2.0, an updated version of the initial bioimpedance algorithm. The updated version is intended to lower the number of false positive alerts. Alterations include a faster changing reference after initialization, a slower accumulating fluid index for an initial duration of the event in patients with higher day-to-day variability in impedance, and a fluid index which accumulates only over the last 30 days [15]. An initial study has reported a 40% decrease in unexplained detections [17]. In our study, the default OptiVol threshold settings were used.

In 2009, St Jude Medical introduced its own bioimpedance algorithm, known as CorVue. There are some fundamental differences compared to the OptiVol algorithms. CorVue also measures intrathoracic impedances in a semicontinuous way every 2 hours around the clock. In addition, depending on the type of lead, the impedance is measured in one (ie, unipolar leads) or multiple vectors. Within the first 2 weeks, the algorithm starts to build a reference impedance which is a long-term moving average (ie, over the last 144 or 168 measurements for CRT or ICD devices, respectively). Afterward, a short-term moving average (ie, over the last 12 measurements) of multivector impedance measurements builds the daily impedance. A bioimpedance alert is triggered when the daily impedance is lower than the reference impedance for a programmable duration known as the congestion trigger (ie, nominal 13 days for ICDs, 14 days for CRT-D, and 16 days for CRT-P) [15]. In our study, the default congestion trigger settings were used.

The presence of a bioimpedance algorithm is dependent on the CIED manufacturer: most Medtronic and St Jude devices are equipped with a bioimpedance algorithm and generate bioimpedance alerts, while Biotronik and Boston Scientific devices do not generate any bioimpedance alerts. In our study, the choice of CIED brand implanted in a particular patient is completely random. Therefore, the presence or absence of a bioimpedance algorithm in this patient population is also randomly assigned.

### Intervention Protocol for Bioimpedance Alerts

All incoming bioimpedance alerts were deemed to be of potential clinical relevance and resulted in a phone contact between the interpreting nurse and the patient. In exceptional cases, where the patient had an in-hospital check-up very recently or had one planned in the near future, a phone contact was not initiated. A custom-made heart failure questionnaire was used to identify potential causes for the bioimpedance alert [14,18]. Additional questions could be asked at the discretion of the health care worker in order to gain better insight. Appropriate feedback and general heart failure education (ie, stress the importance of the conservation of a salt-free diet and fluid restrictions) were always provided. Further action was protocol-driven in consultation with a dedicated heart failure specialist.

### Outpatient Follow-Up

Patients enrolled in remote follow-up visit the outpatient cardiology clinic for device and clinical heart failure follow-up at 6 weeks after implantation and subsequently every 6 months with a minimum of 2 visits per year as per standard practice in our institution. Patients in this study were followed until death, exclusion from remote follow-up, heart transplantation, or February 1, 2015, whichever came first.

### Statistical Analysis

Demographic and functional characteristics were compared using descriptive statistics. Continuous variables are expressed as mean and standard deviation (SD) if normally distributed or median and interquartile range (IQR) otherwise. Survival curves were constructed according to the Kaplan Meier method, with the log-rank test used for comparison among groups. Unadjusted and adjusted hazard ratios (HRs) were calculated by Cox regression analysis with Firth's penalized likelihood correction. To define statistical differences between both groups, the independent samples Student *t* test and Mann-Whitney U test were used for normally and not normally distributed continuous variables, respectively, and the chi-square test and Fisher exact test were used accordingly for categorical variables. To define statistical differences between the different bioimpedance algorithms, the Kruskal Wallis test was used. The significance level for tests was 2-sided with alpha=.05. All statistical analyses were performed using IBM SPSS Statistics 24.0 (IBM Corp); SAS 9.4 (SAS Institute Inc) was used for Cox regression with Firth’s penalization.

## Results

### Study Population

**Figure 1:**
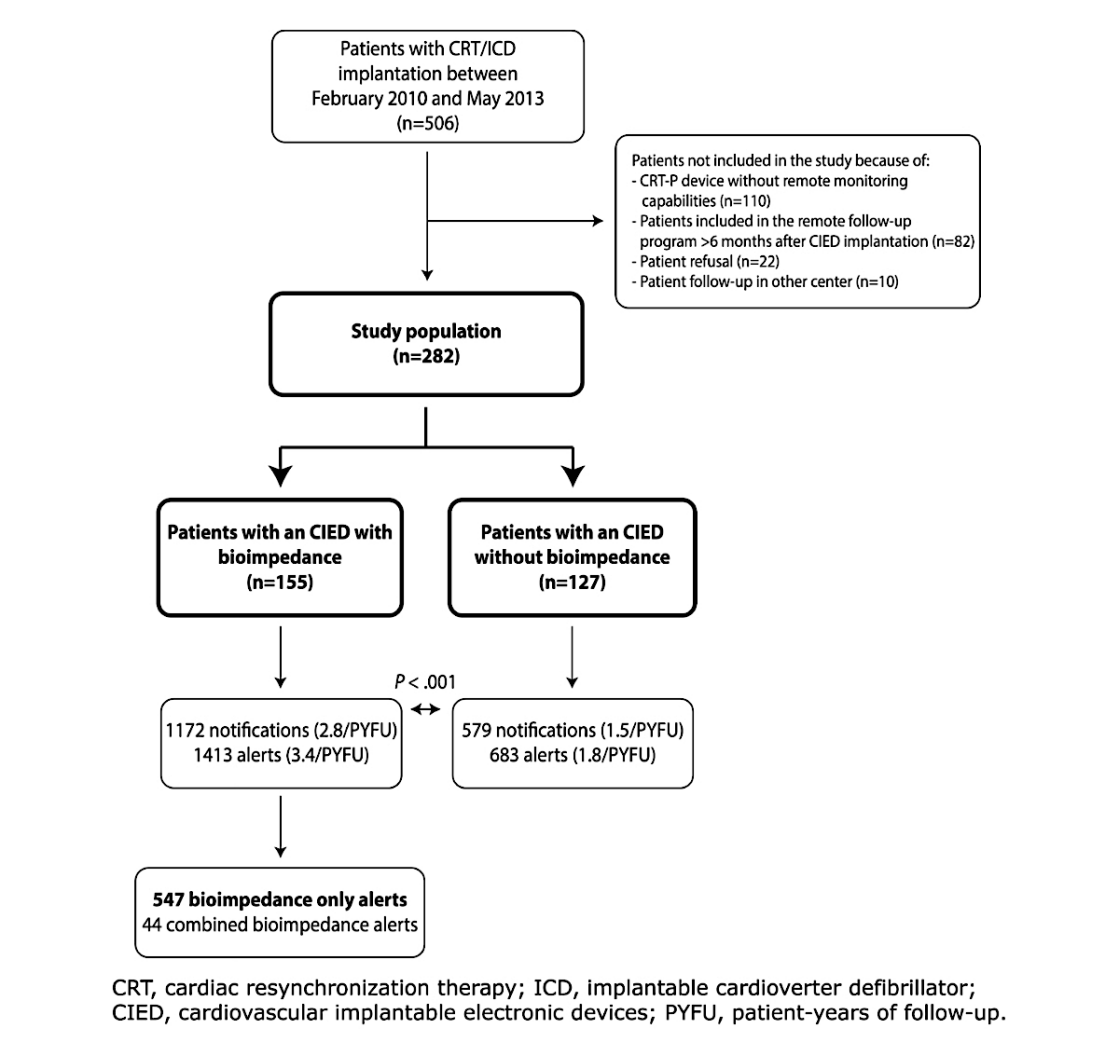
Flowchart of the study.

From a total of 506 patients with a CIED implanted during the study period, 110 patients were excluded due to the presence of a cardiac resynchronization therapy pacemaker (CRT-P) device without remote monitoring capabilities, 82 patients were excluded because the remote monitoring program was started more than 6 months after device implantation, 22 patients refused study participation, and 10 patients were excluded due to follow-up in another center. The final study population consisted of 282 patients: 155 (55.0%) patients with a CIED equipped with a bioimpedance algorithm (CIED^+^) and 127 (45.0%) patients with a CIED without an available bioimpedance algorithm (CIED^—^) (Figure 1). Of 282 devices, 110 (39.0%) Medtronic, 105 (37.2%) St. Jude Medical, 61 (21.6%) Biotronik, and 6 (2.1%) Boston Scientific CIEDs were implanted.

Respectively, 26.4% (41/155), 43.2% (67/155), and 30.3% (47/155) of patients in the CIED^+^ population had a device implanted with Optivol, Optivol 2.0, and CoreVue algorithm. The median time interval between CIED implantation and start of remote follow-up was 1 day (IQR 1 to 2 days), with 81.9% (231/282) of patients included within 1 week. Patients were followed for 34 (SD 12) months leading to 801 cumulative patient-years of follow-up. The number of follow-up visits in the outpatient device clinic was 3.25 per patient-year of follow-up, of which 93% were elective and 7% were triggered by remote monitoring. Baseline characteristics of the study population at the time of implantation are shown in Table 1.

### Remote Follow-Up Notifications, Alerts, and Interventions

**Figure 2:**
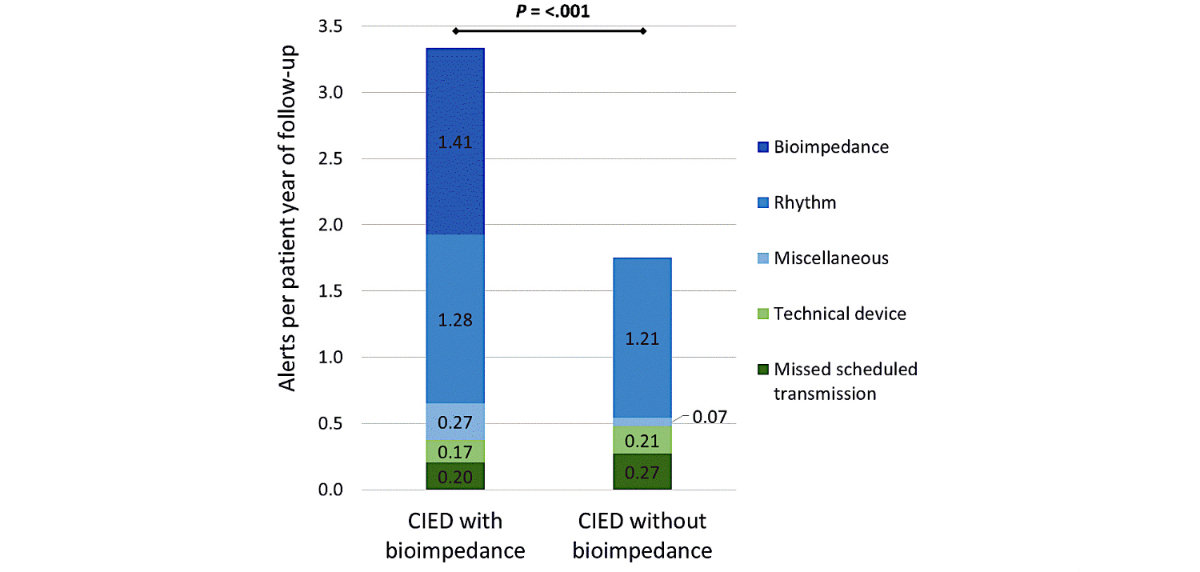
Frequency of alert categories with the number of alerts per patient-year of follow-up for patients with a cardiovascular implantable electronic device with or without a bioimpedance algorithm. Disease-related alerts are marked in blue color tints and technical-related alerts in green color tints.

During follow-up, the clinical call center handled 1751 remote monitoring notifications. Since a notification can contain multiple alerts, a total of 2096 unique alerts were received (ie, 2.6 alerts per patient-year of follow-up). Patients with a CIED^+^ had significantly more alerts than those with a CIED^—^: 1413 (67.41%, 3.4 per patient-year of follow-up) and 683 (32.59%, 1.8 per patient-year of follow-up), respectively, *P*<.001. The amount of technical and arrhythmia alerts was similar in both patient groups. The higher number of alerts in the CIED^+^ population can be entirely attributed to bioimpedance alerts. The distribution of the different alert categories among both groups is shown in Figure 2.

### Bioimpedance Notifications and Interventions

**Figure 3:**
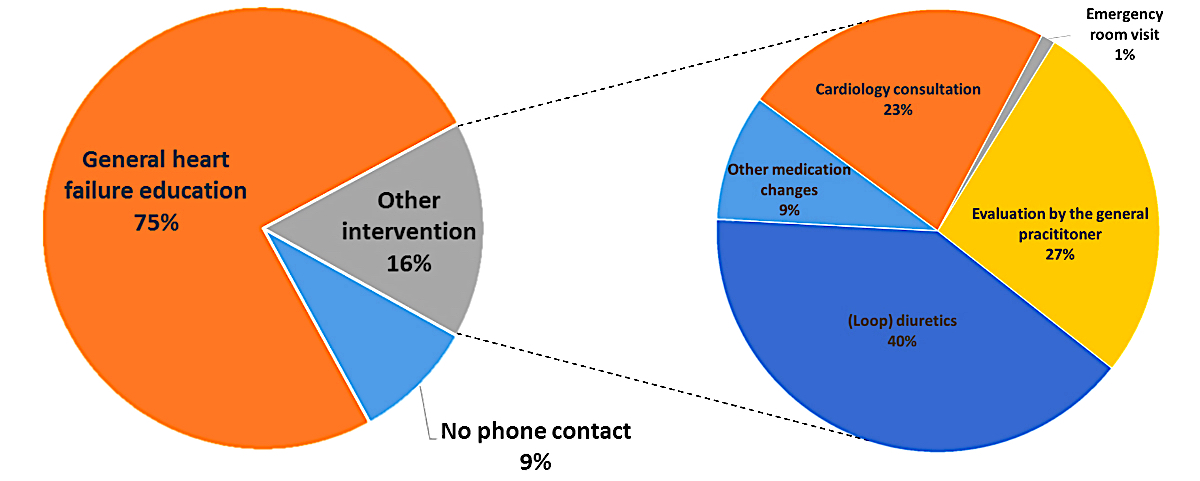
Overview of the interventions triggered during remote follow-up in the case of a bioimpedance-only alert.

During follow-up, 591 notifications including a bioimpedance threshold crossing were received for 111 of 155 (71.6%) patients. In 44 of these notifications, 1 or more additional alerts were combined resulting in 547 bioimpedance-only notifications. In 498 (91.0%) of bioimpedance-only notifications, the patient was contacted by phone and a standardized heart failure questionnaire was used. In 9.0% (49/547) of cases, a phone contact was not initiated since the patient had an in-hospital check-up very recently or had one planned in the near future. In 75.1% (411/547) of bioimpedance-only notifications, only general heart failure education was given. An additional intervention was triggered in 15.9% (97/547) of cases (Figure 3, left). In total, 97 interventions were performed (Figure 3, right), including medication changes in 50% (48/97) of cases, referral to the general practitioner or cardiologist in 27% (26/97) and 23% (22/97) of cases, respectively, and in 1% (1/97) the patient was asked to visit the emergency room. A combination of different interventions for 1 bioimpedance alert is also possible.

**Table 1:** Baseline characteristics of the study population (n=282).

Variables		CIED^a^ with bioimpedance n=155	CIED without bioimpedance n=127	*P* value
Age, years, mean (SD)		72 (12)	70 (13)	.12
BMI^b^, mean (SD)		27 (5)	28 (6)	.54
Male gender, n (%)		123 (79.4)	108 (85.0)	.22
ICD^c^, n (%)		31 (20.0)	59 (46.5)	<.001
CRT-D^d^, n (%)		102 (65.8)	57 (44.9)	<.001
CRT-P^e^, n (%)		22 (14.2)	11 (8.7)	.15
**Bioimpedance algorithm, n (%)**				
	OptiVol	41 (26.4)	—	
	OptiVol 2.0	67 (43.2)	—	
	CorVue	47 (30.3)	—	
**NYHA^f,g^functional class, n (%)**				.42
	Class II	17 (15.6)	14 (23.7)	
	Class III	90 (82.6)	43 (72.9)	
Left ventricular ejection fraction, %, mean (SD)		31 (12)	34 (12)	.01
QRS width, ms, mean (SD)		145 (31)	127 (32)	<.001
**Heart failure etiology, n (%)**				
	Ischemic heart disease	86 (55.5)	86 (67.7)	.04
	Dilated	13 (8.3)	5 (3.9)	.13
	Valvular	3 (1.9)	1 (0.8)	.63
	Hypertrophic	5 (3.2)	3 (2.4)	.73
	Toxic	1 (0.6)	2 (1.6)	.59
	Idiopathic	40 (25.8)	17 (13.4)	.01
	Other etiology or no heart failure	7 (4.5)	13 (10.2)	.06
**Comorbidities and risk factors, n (%)**				
	Valvular surgery	18 (11.6)	11 (8.7)	.42
	Atrial fibrillation	62 (40.0)	51 (40.2)	.98
	Chronic obstructive pulmonary disease	23 (14.8)	16 (12.6)	.59
	Chronic kidney disease	42 (27.1)	31 (24.4)	.61
	Cerebrovascular accident	13 (8.4)	10 (7.9)	.88
	Diabetes	32 (20.6)	25 (19.7)	.84
	Family history of cardiovascular disease	34 (21.9)	40 (31.5)	.07
	Arterial hypertension	59 (38.1)	57 (44.9)	.25
	Hypercholesterolemia	59 (38.1)	48 (37.8)	.96
	Smoking	151 (97.4)	122 (96.1)	.74
**Medication use, n (%)**				
	Renin-angiotensin system blocker	125 (80.6)	102 (80.3)	.94
	Beta-blocker	144 (92.9)	109 (85.8)	.05
	Spironolactone	105 (67.7)	68 (53.5)	.02
	Loop diuretic	76 (49.0)	48 (37.8)	.06
	Digoxin	23 (14.8)	18 (14.2)	.88
	Statin	85 (54.8)	82 (64.6)	.10
	Calcium channel blockers	8 (5.2)	15 (11.8)	.04
	Antidiabetic medication	27 (17.4)	22 (17.3)	.98

^a^CIED: cardiovascular implantable electronic device.

^b^BMI: body mass index.

^c^ICD: implantable cardioverter-defibrillator.

^d^CRT-D: cardiac resynchronization therapy defibrillator.

^e^CRT-P: cardiac resynchronization therapy pacemaker.

^f^NYHA: New York Heart Association.

^g^CIED with bioimpedance group (n=109), CIED without bioimpedance group (n=59).

### Different Bioimpedance Algorithms

**Figure 4:**
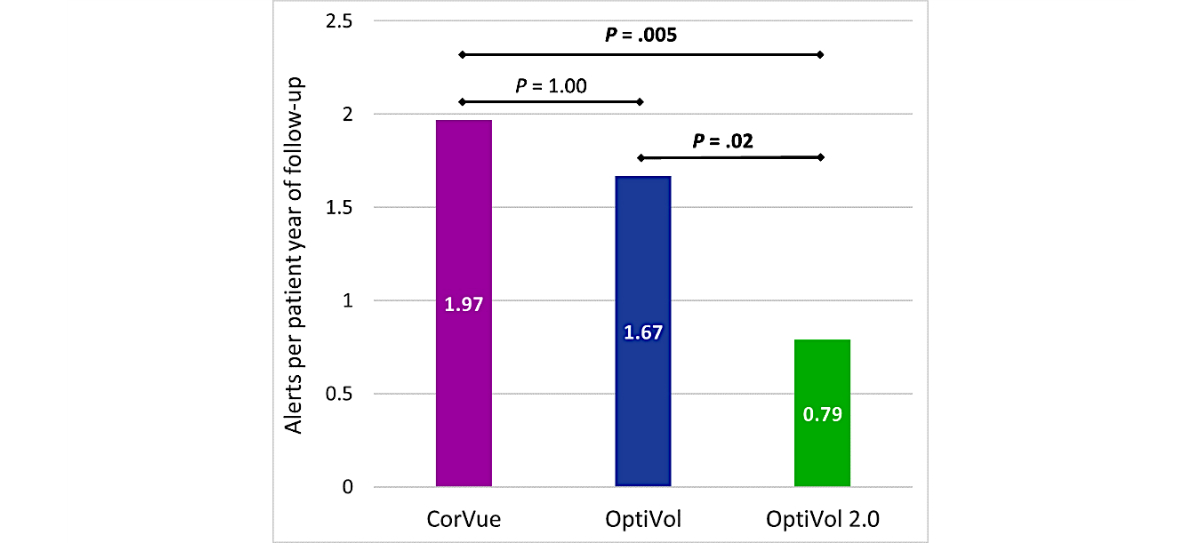
Overview of the amount of remote monitoring bioimpedance alerts per patient-year of follow-up triggered by the different bioimpedance algorithms.

There was a statistically significant difference in number of bioimpedance alerts per patient-year between the different bioimpedance algorithms (χ^2^=12.643, *P*=.002) (Figure 4). The updated OptiVol 2.0 algorithm (0.79 alerts per patient-year of follow-up) triggered significantly fewer bioimpedance alerts than OptiVol (1.67 alerts per patient-year of follow-up; *P*=.02) and CorVue (1.97 alerts per patient-year of follow-up; *P*=.005). No differences were observed concerning the distribution of interventions triggered by the different bioimpedance algorithms.

### Clinical Outcome

**Figure 5:**
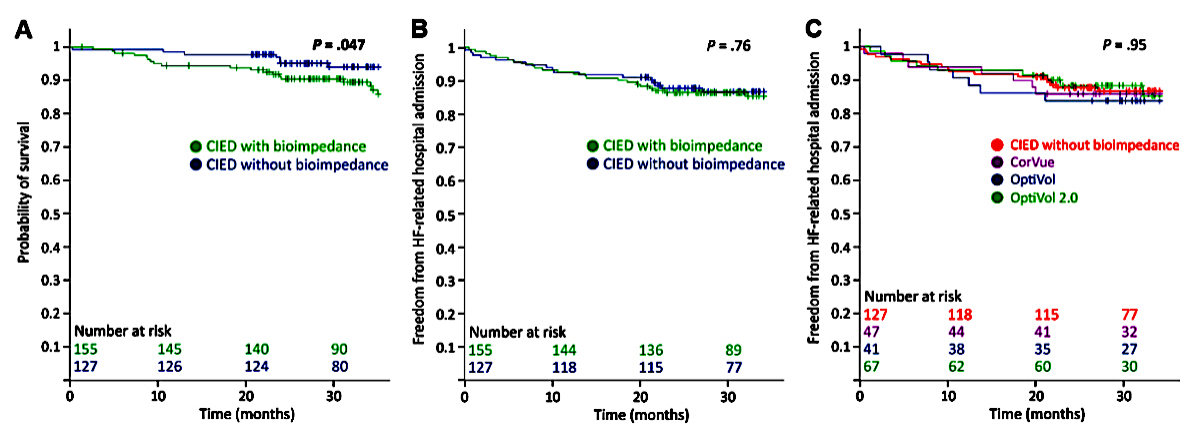
(A) Probability of survival for patients with a cardiovascular implantable electronic device (CIED) with or without a bioimpedance algorithm, (B) Freedom from hospital admission with a primary diagnosis of heart failure for patients with a CIED with or without a bioimpedance algorithm, (C) Freedom from hospital admission with a primary diagnosis of heart failure for the different bioimpedance algorithms.

At mean time of follow-up (ie, 34 months), 26 patients had died, leading to an overall survival rate of 90.8%. Seven patients died in the CIED^—^ population compared to 19 in the CIED^+^ population, leading to all-cause survival rates of 94.5% and 87.7%, respectively (*P*=.047) (Figure 5A). Most deaths were due to cardiovascular causes, with 6 in the CIED^—^ population compared to 15 in the CIED^+^ population (*P*=.10). No significant differences in survival rate were observed for the different bioimpedance algorithms.

At mean time of follow-up, 40 patients were hospitalized with a primary diagnosis of heart failure and hence 85.8% (242/282) of patients were free from heart failure–related hospitalization. No significant difference was observed between both groups (23/155, 85.2%, for CIED^+^ versus 17/127, 86.6%, for CIED^—^ at mean time of follow-up, *P*=.76) (Figure 5B) or for the different bioimpedance algorithms (*P*=.95) (Figure 5C).

No significant differences were observed between both groups with respect to the number of elective follow-up visits in the outpatient heart failure clinic (*P*=.45) or the number of cardiac-related hospital admissions (*P*=.32). For those who had at least 1 cardiac-related hospital admission, median length of hospital stay was 6 (IQR 3 to 14) days. There was no significant difference for length of hospital stay between both groups or for the different bioimpedance algorithms.

**Table 2:** Cox regression analysis with Firth's penalization for clinical outcome measures.

Variables	Unadjusted hazard ratio			Adjusted hazard ratio^a^		
	HR^b^	95% CI	*P* value	HR	95% CI	*P* value
All-cause mortality	2.342	1.029-5.996	.047	2.118	0.845-5.791	.13
Cardiovascular survival	2.168	0.881-6.082	.10	2.335	0.852-7.020	.12
Heart failure hospitalization	1.103	0.592-2.097	.76	1.284	0.655-2.562	.47

^a^Hazard ratios were adjusted for significant differences in baseline characteristics including implantable cardioverter-defibrillator use, cardiac resynchronization therapy defibrillator use, left ventricular ejection fraction, QRS width, ischemic etiology of heart failure and spironolactone use, and clinically relevant parameters including age, gender, and loop diuretic use.

^b^HR: hazard ratio.

Table 2 provides an overview of the unadjusted and adjusted Cox regression analysis with Firth's penalization. Presence of bioimpedance algorithms in the CIED resulted in a nonsignificant adjusted hazard ratio of 2.118 (95% CI 0.845-5.791) for all-cause death and 2.335 (95% CI 0.852-7.020) for cardiovascular death. Multivariate analysis indicated that age, ejection fraction, and QRS time contribute to the observed difference in survival rate between both groups.

## Discussion

### Principal Findings

Although many newly implanted CIEDs have a built-in bioimpedance algorithm, it remains unclear whether bioimpedance measurements contribute to improved clinical outcome when incorporated in a standardized heart failure care path including remote follow-up. In this paper, we present a comprehensive overview of bioimpedance alerts and subsequent triggered interventions in patients with either a CRT or ICD device enrolled in a dedicated, protocol-driven, remote follow-up program in a single Belgian tertiary care center.

Major insights include the following:

1. Patients with a CIED equipped with a bioimpedance algorithm have significantly more remote monitoring notifications
2. In 75% of bioimpedance-only alerts, reenforcing heart failure education was the only action taken; in 16% of cases, an additional intervention was triggered; and in 9%, the patient was not contacted
3. For the different bioimpedance algorithms, significant differences were observed for the number of bioimpedance alerts but not for triggered interventions or clinical outcome

Although patients with a CIED equipped with a bioimpedance algorithm have a significantly lower survival rate, driven by differences in baseline characteristics, there was no difference in heart failure–related hospitalizations.

An important observation that merits further attention is the high number of patients with bioimpedance-only alerts who were contacted and given heart failure education only without any additional interventions. This may indicate a high sensitivity but low specificity of these alerts to detect emerging congestion as well as the existence of a temporal lag between a bioimpedance alert and clinical manifestation of heart failure worsening [5,7-12,19-21]. Therefore, a possible explanation for the rather low number of additional interventions could be that due to these bioimpedance alerts, patients are contacted in the early phase of emerging congestion. By the fact that patients are contacted and general heart failure education is repeated, it is possible that their perception of disease awareness strengthens (ie, importance of fluid and salt restriction, heart failure medication intake, and physical activity), avoiding further worsening of congestion.

The updated OptiVol 2.0 algorithm triggered significantly fewer bioimpedance alerts than the other two bioimpedance algorithms. This corresponds to literature, where a 40% decrease in unexplained bioimpedance alerts was observed [17]. However, no changes in intervention strategy or impact on clinical outcome compared to the other bioimpedance algorithms was observed. Although improvements to bioimpedance algorithms have already been made, this could still indicate that intrathoracic impedance is currently wrongly measured, handled, or interpreted. In the majority of cases, the bioimpedance alarm threshold is set to default. These thresholds should be individually adjusted in order to improve sensitivity and specificity rates, as suggested by previous research [7,8,15]. Another possibility to improve intrathoracic impedance measurements is controlling the circumstances in which measurements are performed. Currently, measurements are performed under different circumstances throughout the day. Since bioimpedance measurements are also influenced by motion and body posture, reliability could be improved by a lower number of measurements that are all performed under the same posture (eg, during the night when lying in a particular posture). Finally, instead of alerts triggered by bioimpedance crossings alone, integration with other parameters currently monitored by implantable electrical devices (eg, patient activity, heart rate variability, average ventricular rate) will provide a more efficient tool to predict heart failure worsening [18].

Patients with a CIED^+^ showed a lower overall survival rate. However, this difference can be explained by differences in baseline characteristics between both groups rather than the presence of a bioimpedance algorithm. Indeed, multivariate analysis indicated a significant hazard ratio for age, ejection fraction, and QRS time and a nonsignificant adjusted hazard ratio for the presence of a bioimpedance algorithm in the CIED. Concerning heart failure–related hospitalizations, both groups showed similar results. This could indicate a potential benefit of bioimpedance algorithms on clinical outcome since, based on baseline characteristics, one would expect a higher number of heart failure–related hospitalizations in the CIED^+^ group. This could mean that the high frequency of heart failure education provided in cases of a bioimpedance alert could have prevented heart failure–related hospitalizations.

When reviewing available literature, it is clear that the success rate of remote monitoring is strongly dependent on optimal workflow with standardized protocols and appropriate feedback loops. The DOT-HF (Diagnostic Outcome Trial in Heart Failure) trial [9], where patients received an audible alert in case of a bioimpedance crossing, showed that providing wrong feedback can even induce an increase in hospital admissions. In the LIMIT-CHF (Lung Impedance Monitoring in Treatment of Chronic Heart Failure) trial [15], bioimpedance alerts triggered empirical changes in diuretic dose, which did not significantly prevent heart failure–related hospitalizations. Moreover, the OPTILINK-HF (Optimization of Heart Failure Management using OptiVol Fluid Status Monitoring and CareLink) trial [12] employed a similar approach of protocol-driven remote monitoring as used in our center and reported no significant improvements in clinical outcome. Shortcomings in this trial were the single-parameter follow-up, suboptimal data transmission, and the absence of a centralized monitoring team. In our study, a multiparameter approach was used and all incoming alerts were handled in a standardized way. In addition, remote follow-up in our center is performed by a small team of dedicated heart failure nurses who have close personal contact with the patients. Furthermore, our nurses are operating from inside our tertiary care center and hence have daily contact with the treating physician. This approach facilitates protocol standardization and has already been shown to be effective in the IN-TIME (Influence of Home Monitoring on Mortality and Morbidity in Heart Failure Patients With Impaired Left Ventricular Function) trial [22]. It is clear that there is not just one remote monitoring approach, but a high variability exists and hence each approach needs to be assessed on its individual merit. In our study, the question remains to what extent reinforcement of heart failure education is crucial in remote bioimpedance monitoring and impacts clinical outcome.

### Study Limitations

This study should be interpreted in the light of some limitations. First, this is a relatively small single center observational registry study with classic limitations associated with this type of study design, thereby making the study results mainly hypothesis-generating. Although a nonsignificant adjusted hazard ratio was obtained for the presence of bioimpedance algorithms in the CIED, a potential power problem can be present in the all-cause survival analysis due to the rather low sample size and low event rates. Next, since the presence of a bioimpedance algorithm is dependent on CIED manufacturer and is therefore random, our study is a nonrandomized clinical trial. A possible selection bias could be present due to the device indication. However, the assignment of the type of CIED that was implanted was solely based on the device’s therapeutic capabilities (right ventricular pacing, biventricular pacing, and antitachycardia treatment) and was left to the discretion of the treating physician. Other diagnostic information, for example, the presence or absence of bioimpedance algorithms in the CIED, was not taken into consideration when assigning the CIED type or brand. In the CIED^+^ population, more CRT-D devices are present since these devices were first equipped with bioimpedance algorithms. In general, patients who receive a CRT-D device have more advanced heart failure than patients who receive an ICD device. This can explain the differences in baseline characteristics (eg, older population with a lower left ventricular ejection fraction and broader QRS complex). Finally, study inclusion was based on voluntary participation to remote follow-up. Therefore, one cannot exclude the possibility that enrolled patients were more motivated for follow-up with better expected compliance to therapies. However, the majority (>95%) of patients agree to remote follow-up, reducing the risk for selection bias.

### Conclusion

In patients with a CIED with a bioimpedance algorithm, bioimpedance alerts constitute almost half (42%) of incoming alerts when turned on during remote follow-up. Repeating general heart failure education by phone sufficed in 75% of cases, and an additional intervention was performed in 16% of cases. The high frequency of heart failure education that was provided could have contributed to fewer heart failure–related hospitalizations despite significant differences in baseline characteristics. Future trials are needed to verify whether bioimpedance algorithms can only be used to trigger heart failure education or if they have an intrinsic value to change treatment strategies. In addition, future improvements in the way bioimpedance is measured, handled, or interpreted could further increase its clinical relevance. Before bioimpedance measurements can be widely implemented in clinical practice, larger multicenter randomized controlled trials are required.

## Abbreviations

CRT: cardiac resynchronization therapy
CRT-D: cardiac resynchronization therapy defibrillator
CRT-P: cardiac resynchronization therapy pacemaker
DOT-HF: Diagnostic Outcome Trial in Heart Failure trial
ICD: implantable cardioverter-defibrillator
CIED: cardiovascular implantable electronic device
CIED^+^: cardiovascular implantable electronic device with bioimpedance algorithm
CIED^—^: cardiovascular implantable electronic device without bioimpedance algorithm
IN-TIME: Influence of Home Monitoring on Mortality and Morbidity in Heart Failure Patients With Impaired Left Ventricular Function trial
IQR: interquartile range
HR: hazard ratio
LIMIT-CHF: Lung Impedance Monitoring in Treatment of Chronic Heart Failure trial
OPTILINK-HF: Optimization of Heart Failure Management using OptiVol Fluid Status Monitoring and CareLink trial
SD: standard deviation
